# Pulmonary Mucormycosis: A Case Report of a Rare Infection with Potential Diagnostic Problems

**DOI:** 10.1155/2020/5845394

**Published:** 2020-01-06

**Authors:** Salwa O. Mekki, Amal A. Hassan, Afnan Falemban, Nashwa Alkotani, Salem M. Alsharif, Ahmed Haron, Basim Felemban, Mohammad S. Iqbal, Aisha Tabassum

**Affiliations:** ^1^Pathology Department, Alnoor Specialist Hospital, Makkah, Saudi Arabia; ^2^Faculty of Medicine, Al Neelain University, Khartoum, Sudan; ^3^Faculty of Medicine, Al-Azhar University, Cairo, Egypt; ^4^Department of Medicine, Alnoor Specialist Hospital, Makkah, Saudi Arabia; ^5^Medical Department, Alnoor Specialist Hospital, Makkah, Saudi Arabia; ^6^Radiology Department, Alnoor Specialist Hospital, Makkah, Saudi Arabia; ^7^Department of Pathology, Faculty of Applied Medical Sciences, Umm Al Qura University, Makkah, Saudi Arabia

## Abstract

Pulmonary mucormycosis is a relatively rare pulmonary fungal disease, which is difficult to diagnose early and lacks effective treatment. It is seen in patients with hematological malignancies, diabetes mellitus, and immunocompromised states. The diagnosis depends primarily on the detection of fungi in lung tissue. Here, we present a case of a 52-year-old male who has type 2 diabetes mellitus and a past history of treated pulmonary tuberculosis. Clinical diagnosis is difficult in pulmonary mucormycosis, and early diagnosis is needed for this life-threatening infection. Histopathological examination of a resected cavity confirmed the diagnosis of pulmonary mucormycosis. This report highlights the difficulty of diagnosis and the importance of histological examination in detecting mucormycosis which will help for early management.

## 1. Introduction

Mucormycosis is a rare opportunistic fungal infection. It derives its name from the *Mucorales* order of filamentous fungi and family *Mucoraceae*. It is a less common opportunistic fungal infection compared to *Candida* and *Aspergillus* species [1]. There are six most commonly reported forms which include rhinocerebral, pulmonary, cutaneous, gastrointestinal, disseminated, and uncommon presentations [2, 3]. The relative incidence of pulmonary mucormycosis (PM) to other clinical form incidence in literature is about 25% [4]. Diabetes mellitus, systemic corticosteroid therapy, neutropenia, hematologic malignancies, stem cell transplant, and immunocompromised state are the predisposing situations for mucormycosis [3, 4]. Pulmonary mucormycosis results from the inhalation of sporangiospores or by hematogenous or lymphatic spread [3, 5]. Patients present with nonspecific symptoms like cough, dyspnea, chest pain, and fever [3, 5]. Clinical diagnosis is difficult in pulmonary mucormycosis, and early diagnosis is needed for this life-threatening infection [6]. There are no reliable serological, PCR-based, or skin tests for mucormycosis. Sterile culture does not rule out the diagnosis. Histopathology and direct microscopy along with culture from various clinical specimens except blood are the major diagnostic modalities for mucormycosis [5].

Here, we present a case of pulmonary mucormycosis arising in a treated case of pulmonary tuberculosis.

## 2. Case Report

**Figure 1:**
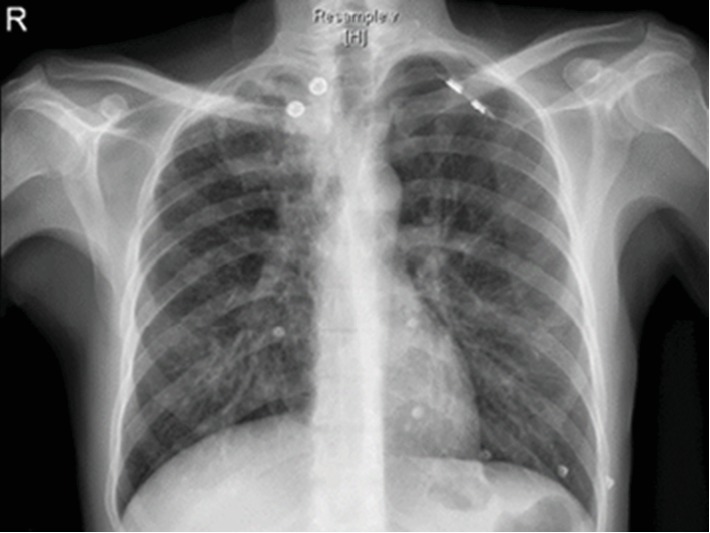
Chest radiograph, PA view showing cavitary lesion in the right upper lobe.

**Figure 2:**
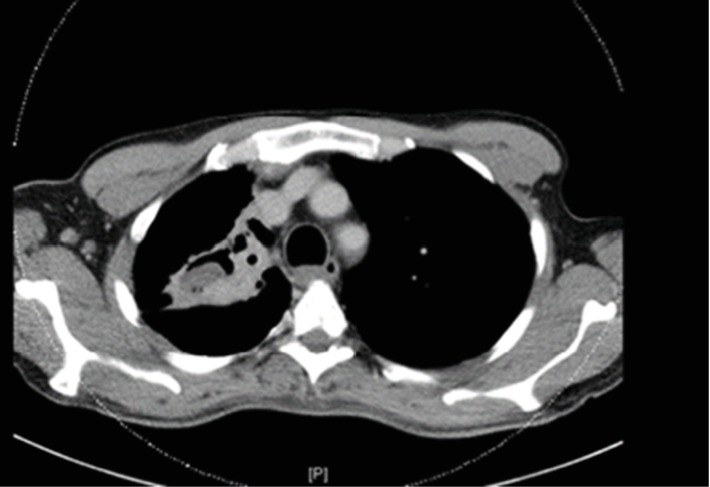
CT showing cavitation and partial collapse of the right upper lobe.

A 52-year-old male patient, known diabetic, presented to the emergency department with a complaint of hemoptysis. There was no fever, chest pain, or bleeding from other sites. No history of weight loss or night sweats. The patient is an old treated pulmonary tuberculosis case. There i no history of any drug intake. On admission, the patient was stable. General physical examination was insignificant. Local examination revealed upper lobe crepitation. CT chest angiography and chest radiograph (Figures 1 and 2) revealed right upper lobe partial collapse and old healed pulmonary tuberculosis of the right upper lobe with underlying cavitation and bronchiectasis. A well-defined, hypodense, nonenhancing lesion is noted in the cavitation that was reported as a mucous plug or a fungus ball. Right bronchial arteries were hypertrophied and tortuous. There was no endobronchial lesion or mucosal abnormalities. The right bronchial tree showed minimal hemorrhage. There was a cavity in the right apex. There were no aneurysms or vascular malformations. No significant changes were associated with bronchiectatic changes. Bronchoalveolar lavage analysis showed inflammatory cells, macrophages, and degenerated cells. There was no evidence of malignancy. Culture was done for bronchoalveolar lavage, and no growth was identified. Direct microscopic examination was not done.

**Figure 3:**
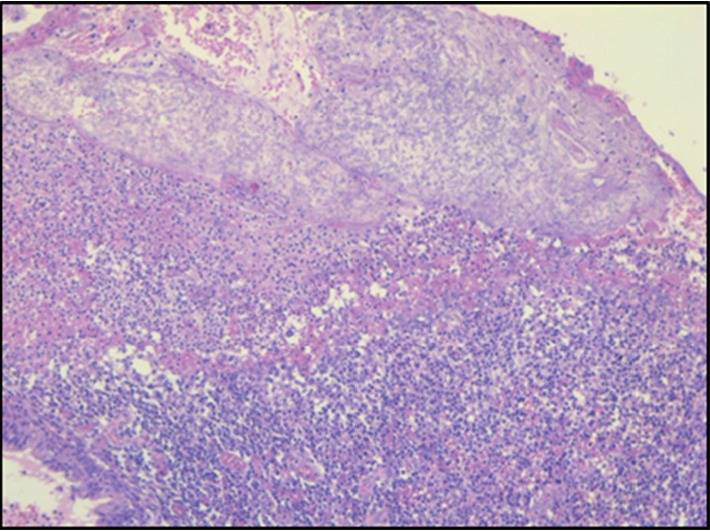
Lung tissue with cavity containing fungal ball (arrow), H&E 20x.

**Figure 4:**
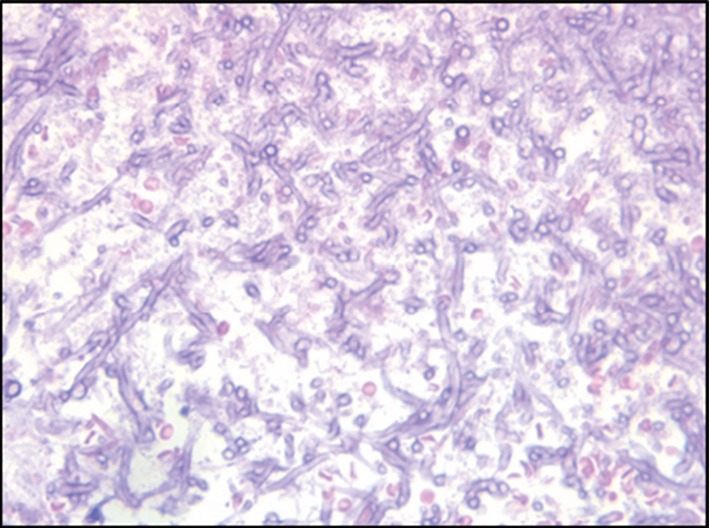
Broad nonseptate hyphae with right-angled branching, H&E 40x.

**Figure 5:**
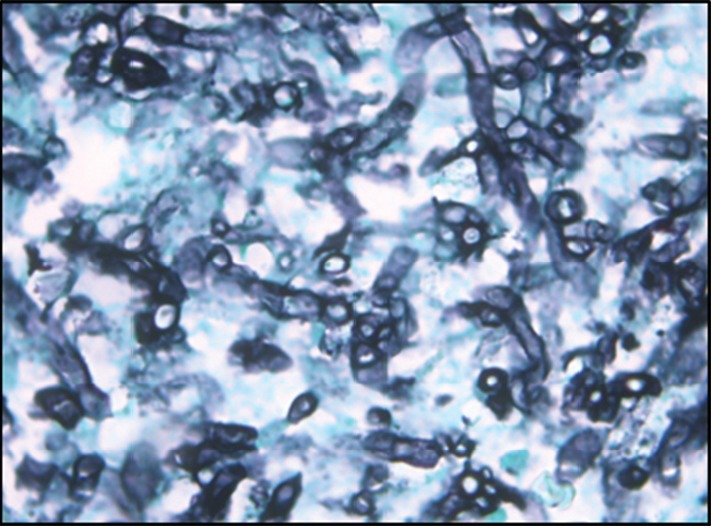
Broad nonseptate hyphae with right-angled branching, Gomori's methenamine silver (GMS), 100x.

**Figure 6:**
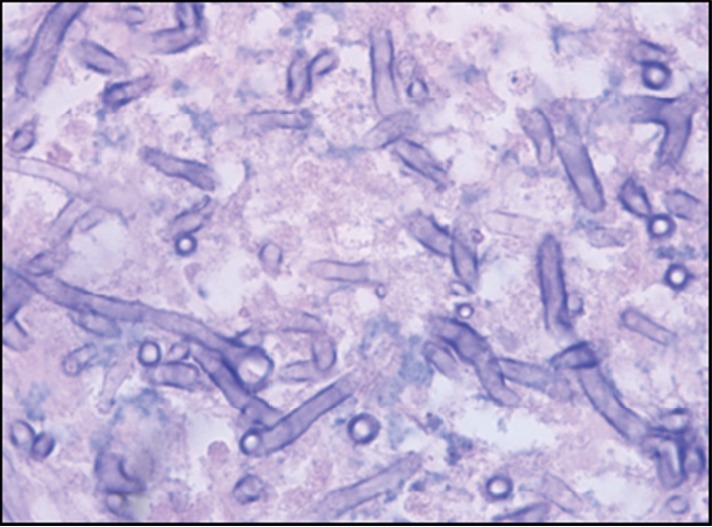
Broad nonseptate hyphae with right-angled branching, H&E 100x.

CT angiography and right bronchial artery shunt embolization was performed. After embolization, the patient was stable, and no more complaints of hemoptysis were reported. The patient was discharged and put on voriconazole 200 mg per oral q12h and levofloxacin 500 mg per oral q24h. Four days after discharge, the patient came to the emergency department with complaint of hemoptysis. On examination, the patient was vitally stable. Right upper lobe crepitations were present. General physical examination was unremarkable. Laboratory investigations showed a hemoglobin of 10 gm/dl, total WBC count of 19,300 cells/cumm, and platelets 4.02 lakhs/cumm. Biochemical investigations revealed a BUN of 5.71 mmol/l and creatinine 75 *μ*mol/l. The thoracic surgery unit was consulted, and they took the patient for surgical management. Thoracotomy and limited resection of the lung lesion including the fungal ball from the right upper lobe was done, and the sample was sent for pathological examination. Microscopic examination of lesion revealed lung tissue filled with colonies of broad, nonseptate right-angled branching fungal hyphae. Gomori's methenamine silver stain highlighted the fungi with spores. There were many epithelioid cells admixed with mixed inflammatory infiltrates. A diagnosis of pulmonary mucormycosis was done on histopathology (Figures 3–6). Postsurgery culture was negative. After confirmation, voriconazole was changed to liposomal amphotericin B 5 mg/kg IV q24h for total 6 weeks. The patient could not be followed up later.

## 3. Discussion

Mucormycosis encompasses a group of infections caused by the fungi belonging to the order *Mucorales* and family *Mucoraceae* [1, 7]. *Rhizopus oryzae* is the most common cause of infection from the *Mucoraceae* family followed by *Mucor* sp. and *Lichtheimia* sp. Inhalation of ubiquitous spores is the main mode of infection. There is no evidence of human to human transmission [8]. Recent reclassification has abolished the class *zygomycetes*, and hence, the term zygomycosis is inappropriate for use, although it may still be used to address mucormycosis [1]. Pulmonary mucormycosis is a devastating and life-threatening infection if not correctly diagnosed and treated [9]. The mononuclear and polymorphonuclear phagocytes of normal hosts kill Mucorales by generation of oxidative metabolites and the cationic defensins. Defensive mechanisms in normal hosts are mediated by macrophages that inhibit germination of spores and neutrophils that kill hyphal elements by oxidative burst. Macrophage dysfunction is the cause for infection in diabetes patients [10]. Weak host defenses are a major risk factor for pulmonary mucormycosis. Neutropenic patients as well as patients with elevated serum iron levels are also at increased risk of developing mucormycosis [5].

Pulmonary mucormycosis may develop as a result of inhalation of spores or by hematogenous or lymphatic spread. Portal of entry for *Mucorales* is the respiratory tract where the fungi can easily invade arteries, veins, and lymphatics and produce thrombosis and infarction which can be fatal [11–13].

Invasive pulmonary mucormycosis causes severe morbidity and mortality in patients with hematological malignancies, diabetes mellitus, hematopoietic stem cell transplant and solid organ transplant patients, patients on corticosteroid-based therapy, iron overload and chelation therapy, intravenous drug use, trauma, burns, neonatal prematurity, and malnutrition [14–19]. The sequelae include angioinvasion and direct tissue injury of the respiratory tract, direct extension from the lungs into the great vessels, invasion from the paranasal sinuses into the orbit and brain, and hematogenous dissemination to the central nervous system [14, 20]. Clinical presentations may include nonspecific symptoms like fever, dyspnea, cough, and chest pain, and rarely, they can present as progressive subcutaneous emphysema, Pancoast syndrome, Horner's syndrome, and bronchial perforation [21]. Angioinvasion results in necrosis of parenchymal tissue, cavitation and/or hemoptysis which may be fatal if a major blood vessel is involved [22].

In a large review of 929 cases of zygomycosis, an overall mortality of 44% was reported in diabetics with zygomycosis and 76% mortality for pulmonary zygomycosis in patients with other predisposing factors. *Rhizopus* species were the most common organism in patients of zygomycosis in whom hematological malignancies were the predisposing factor [16].

Histopathology and direct microscopy along with culture for various clinical specimens are the major diagnostic modalities for mucormycosis. Sputum and BAL cytology are unpredictable and may be negative [10, 23]. Histopathologically, mucormycosis species appear as broad, nonseptate hyphae with right angle branching and can be differentiated from Aspergillus which shows regular, septate, and acute angle branching hyphae [24].

Radiological findings described in literature are the presence of nodule or consolidation, isolated mass, cavitation, or lung abscess with fungal ball. Wedge-shaped infarcts of the lung may also be seen, particularly following thrombosis of the pulmonary vessels due to fungal angioinvasion. High-resolution CT scan is the best method of determining the extent of pulmonary mucormycosis and may demonstrate evidence of infection. Expansion of a mass or consolidation across tissue planes towards the great vessels in the mediastinum may suggest the diagnosis [25, 26]. “Reverse halo sign” is described as a prominent CT finding present in the early course of disease [5].

Differential diagnosis of PM involves bacterial, viral, and other fungal pneumonias and invasive fungal infections can be a diagnostic challenge [27]. Invasive PM needs to be distinguished from invasive aspergillosis as the two respond differently to antifungal agents [24]. Confirmation by microscopic examination is necessary as treatment will differ. In our case, before confirmation by histopathology, the patient was put on voriconazole, and after confirmation, it was changed to liposomal amphotericin B. Rarely, PM may recur even in an immunocompetent host [28]. Lipid formulation of amphotericin B is the first-line therapy for mucormycosis [29].

## 4. Conclusion

Mucormycosis is an opportunistic life-threatening infection mostly occurring in the immunocompromised host. The risk factors are many including hematological malignancies, uncontrolled diabetes mellitus, and immunocompromised states. Rhino-orbito-cerebral is the most predominant form of clinical presentation. Diagnosis is difficulty due to nonspecific presentation. Early diagnosis and treatment which includes surgery and antifungal drugs can improve outcome and survival. Definitive diagnosis required pathologic demonstration of the organism in affected tissue as sputum and BAL culture rarely shows growth. The best promising diagnostic modalities should be applied at the earliest to avoid unnecessary treatment with voriconazole and promoting early specific management of mucormycosis.
